# Training-induced cognitive and neural plasticity

**DOI:** 10.3389/fnhum.2013.00048

**Published:** 2013-02-22

**Authors:** Julia Karbach, Torsten Schubert

**Affiliations:** ^1^Department of Educational Science, Saarland UniversitySaarbruecken, Germany; ^2^Department of Psychology, Humboldt UniversityBerlin, Germany

Research on cognitive interventions and training-induced changes in brain and behavior has been of growing interest in cognitive neuroscience and related disciplines over the last decade (for reviews see Hertzog et al., 2008; Lustig et al., 2009; Shipstead et al., 2010; Morrison and Chein, 2011; for a recent meta-analysis see Melby-Lervåg and Hulme, 2013). The aim of this research topic is to provide a broad scope of state-of-the art research in order to advance the understanding of the scope and the mechanisms involved in cognitive and neural plasticity, that is, the potential modifiability of a person's cognitive abilities and brain activity.

Previous studies focusing on the magnitude and maintenance of training-related benefits have indicated that plasticity is considerable in healthy individuals across lifespan (e.g., Brehmer et al., 2007; Karbach and Kray, 2009; Karbach et al., 2010; Dorbath et al., 2011; Strobach et al., 2012a,c), and that it may even extend to very old age (Verhaeghen et al., 1992; Buschkuehl et al., 2008; Zinke et al., 2012b). Aside from training-related improvements on the trained task, researchers are especially interested in understanding the transferability of training-related performance gains to tasks that have not been part of the training. This issue is of particular importance for the application of training programs, e.g., in clinical and educational contexts, but also for the theoretical understanding of the processes underlying training and transfer effects. Recent evidence indicated that transfer effects might be enhanced if the training regime taps higher-level executive control processes instead of focusing on basic processing commodities or specific strategies (Lustig et al., 2009; Noack et al., 2009). Others showed that transfer of training can only occur if the training task and the transfer task engage overlapping cognitive processing components and brain regions (Dahlin et al., 2008). In addition, findings from behavioral cognitive training research have been accompanied by findings from cognitive neuroscience, indicating that cognitive training often induces practice-related changes in the neural substrate (for reviews see; Kelly and Garavan, 2005; Jones et al., 2006; Klingberg, 2010). These observations point to training-induced plasticity in several cortical and subcortical regions which can relate to neural changes within these regions as well as in networks of regions, emphasizing the importance of interdisciplinary approaches for investigating cognitive and neural changes after training.

The contributions of this research topic have addressed the nature, the scope and the preconditions of cognitive and neural plasticity from different angles. Two review articles provide an overview of recent findings on cognitive training in the areas of developmental psychology (Jolles and Crone, 2012) and cognitive aging (Buitenweg et al., 2012). Cognitive plasticity in childhood and in older age has also been addressed by several original research articles (Brehmer et al., 2012; Garrett et al., 2012; Hanna-Pladdy and Gajewski, 2012; Kray et al., 2012; Lövdén et al., 2012; Lussier et al., 2012; Söderqvist et al., 2012; Strobach et al., 2012b; Zinke et al., 2012a). The findings reported in these publications provide strong evidence for the view that cognitive plasticity extends from childhood to older age (c.f. Brehmer et al., 2007; Karbach and Kray, 2009). Moreover, these results are supported by evidence indicting that cognitive plasticity is not only present in healthy individuals, but can also be found in patients suffering from developmental disorders (Kray et al., 2012), intellectual disability (Söderqvist et al., 2012), and chronic traumatic brain injury (Sacco et al., 2011).

In addition to investigating the effectiveness of cognitive training in different populations, such as different age groups or different types of patients, several contributions have also provided evidence for the usefulness of different training regimes. Most of these studies applied process-based training interventions, such as executive-control training (Kray et al., 2012; Lussier et al., 2012; Strobach et al., 2012b), working-memory training (Brehmer et al., 2012; Salminen et al., 2012; Schneiders et al., 2012; Söderqvist et al., 2012) or game training (Prakash et al., 2012; van Muijden et al., 2012), but also different types of physical training (Gajewski and Falkenstein, 2012; Zinke et al., 2012a,b). Nevertheless, it remains open which of these kinds of training most efficiently support the occurrence of transfer effects. Consistent with the growing interest in understanding the neural mechanisms underlying training-induced performance changes, a few of the studies have also applied neurophysiological (Gajewski and Falkenstein, 2012) und neuroimaging techniques (Sacco et al., 2011; Prakash et al., 2012; Schneiders et al., 2012), suggesting that training-induced behavioral changes were accompanied by significant changes in neural activity that varied as a function of the specific training intervention.

Recently, it has also been suggested to analyze training data from an individual differences perspective (see also Garrett et al., 2012). Addressing the question why some individuals benefit more than others from cognitive interventions is particularly important for the adaptation of training regimes to populations with specific needs. Two articles (Buitenweg et al., 2012; Jolles and Crone, 2012) have pointed to the importance of this aspect and Lövdén et al. (2012) have reported significant individual differences in memory training and transfer effects across the lifespan. However, a minimum cognitive capacity seems a necessary precondition for the manifestation of training and transfer effects (Söderqvist et al., 2012).

In sum, the current research topic provides a broad overview of new findings and contributes to a deeper understanding of cognitive and neural plasticity. It shows cognitive training to be a promising tool for investigating basic mechanisms of adaptive behavior and neuronal functioning as well as for designing training applications and interventions. The current findings have also pointed to a number of important topics and unsolved issues that will be relevant for forthcoming research: Among them questions regarding methodological approaches in training research, the mechanisms mediating the transfer of training-related benefits, and the usefulness of training for enhancing activities of daily living in different clinical and non-clinical populations.
